# The Three Dimensional Spatial Structure of Antarctic Krill Schools in the Laboratory

**DOI:** 10.1038/s41598-018-37379-9

**Published:** 2019-01-23

**Authors:** David W. Murphy, Daniel Olsen, Marleen Kanagawa, Rob King, So Kawaguchi, Jon Osborn, Donald R. Webster, Jeannette Yen

**Affiliations:** 1 School of Civil and Environmental Engineering, Georgia Institute of Technology, Atlanta, GA 30332 USA; 2 Department of Mechanical Engineering, University of South Florida, Tampa, FL 33620 USA; 3 Australian Antarctic Division, Kingston, Tasmania 7050 Australia; 4 Discipline of Geography and Spatial Sciences, School of Technology, Environments and Design, University of Tasmania, Hobart, Australia; 5 School of Biological Sciences, Georgia Institute of Technology, Atlanta, GA 30332 USA

## Abstract

Animal positions within moving groups may reflect multiple motivations including saving energy and sensing neighbors. These motivations have been proposed for schools of Antarctic krill, but little is known about their three-dimensional structure. Stereophotogrammetric images of Antarctic krill schooling in the laboratory are used to determine statistical distributions of swimming speed, nearest neighbor distance, and three-dimensional nearest neighbor positions. The krill schools swim at speeds of two body lengths per second at nearest neighbor distances of one body length and reach similarly high levels of organization as fish schools. The nearest neighbor position distribution is highly anisotropic and shows that Antarctic krill prefer to swim in the propulsion jet of their anterior neighbor. This position promotes communication and coordination among schoolmates via hydrodynamic signals within the pulsed jet created by the metachronal stroking of the neighboring krill’s pleopods. The hydrodynamic communication channel therefore plays a large role in structuring the school. Further, Antarctic krill avoid having a nearest neighbor directly overhead, possibly to avoid blockage of overhead light needed for orientation. Other factors, including the elongated body shape of Antarctic krill and potential energy savings, also may help determine the three dimensional spatial structure of tightly packed krill schools.

## Introduction

Benefits of collective behavior among animal groups such as fish schools and bird flocks may include protection from predators^1^, heightened alertness to predators and food resources^2,3^, and lower energetic cost of transport^4–8^. The positions that moving animals take up relative to conspecifics potentially can reveal a sought-after benefit of collective motion. Drafting behind a conspecific, for example, has been shown to save energy in queues of spiny lobsters and lines of ducklings^4,9^. Large birds flying in the arms of a V formation and thus in the upwash created by their anterior neighbor have similarly been shown to use less energy compared to flying alone^6,8^. Maintaining these positions and coordinating movement among conspecifics require numerous, continuous interactions among group members as they individually respond to cues provided by their neighbors^10^. These cues may include the position, speed, acceleration, or movement direction of one or several neighbors. Furthermore, these cues may be mediated by single or multiple sensory modalities including sound, vision, mechanoreception, and chemoreception^11–14^. Mechanoreception may include both direct contact with neighbors^4^ and fluid flow signals encoded in quantities such as flow magnitude, acceleration, or strain rate^15–17^. Such cues then may prompt an animal to change its own speed, heading, or position relative to a neighbor.

Antarctic krill (*Euphausia superba*) are a keystone species of the Southern Ocean and, as 3–5 cm long adults, are highly social obligate schoolers^18–20^. A typical krill school has a length of 100 m, width of 3–4 m, and depth of 10 m, and school densities have been visually estimated at 20,000 to 30,000 individuals per cubic meter^21^. Krill schools have been observed migrating up to 12 km per day over several days^22^ and, upon finding food, will break up into an unorganized swarm to feed^23^. Schooling is thus a key behavior for Antarctic krill, but many questions about communication among schoolmates and the potential benefits of schooling remain. For example, vision, olfaction, and hydrodynamic signals created by krill swimming are all thought to contribute to school maintenance^12,16,17,21,24^. The relative importance of these sensory modalities and how their importance might change under different environmental conditions (i.e. light vs. dark, high vs. low predation risk) is not known. In addition, schooling is thought to be a generalized anti-predator strategy for Antarctic krill^19^, but reduced cost of locomotion also has been suggested as a possible benefit^25–27^. Answers to these questions require detailed examination of the species’ sensory capabilities, the flow fields generated by Antarctic krill, and the positions that Antarctic krill take up relative to schoolmates.

O’Brien pioneered the study of the structure of Antarctic krill in schools in the laboratory and found that nearest neighbor distance increased with increasing light levels and decreased with predator presence^28^. However, the small number of krill positions measured limited this study. Further, the unwarranted conclusion that krill avoided positions above and below neighbors was reached because the nearest neighbor elevation distributions were not normalized (by the Jacobian factor) to account for the fact that nearest neighbor elevation distributions are not constant even in a random aggregation^29,30^. Catton *et al*. measured the two dimensional (i.e. projected) positions of krill swimming in small groups and found many nearest neighbors above or below the focal krill, but too few measurements were acquired to draw firm conclusions^17^. Several other recent studies also have measured similarly small numbers of nearest neighbor distances or krill positions^31–33^. In the current study, we examine the three dimensional structure of Antarctic krill schools in the laboratory. By using a sufficiently large number of observations of krill positions within schools and by taking into account the current state of knowledge on krill sensory capabilities and the flow fields they generate, we aim to discuss hypotheses regarding the benefits and interaction rules of schooling in Antarctic krill.

## Methods

Antarctic krill schooling data were collected at the Australian Antarctic Division (AAD) in Kingston, Tasmania, where Kawaguchi *et al*. demonstrated that krill regularly could be induced to school for at least a year after capture^31^. The krill under study here were captured at 100 m depth by the *Aurora Australis* via rectangular midwater trawl on March 24, 2007 at 66° 04.13′ S, 109° 58.69′ E, arrived at the AAD on April 1, 2007, and were filmed in the austral winter of 2008. Several hundred krill were housed in an 1860 liter cylindrical holding tank with a diameter of 1.432 m and were cared for as described in Kawaguchi *et al*.^31^. Krill regularly demonstrated schooling behavior throughout the course of the study. Murphy *et al*. studied the swimming kinematics of the same krill population and found a mean body length *L*_1_ = 34 ± 9 mm (mean ± standard deviation)^34^, measured from the telson tip to the front of the eyeball. Including the antennules in the length measurement (estimated at approximately 12 mm from high speed videos), as in Catton *et al*., extends the mean krill body length to a nominal krill length of *L*_2_ = 46 mm^17^. Body lengths *L*_1_ and *L*_2_ are differentiated here because antennules may be lost or truncated and may be actively pointed in different directions. The measurement *L*_1_ is thus a better indicator of body length relating to swimming speed, and *L*_2_ is a better indicator of body length relating to spacing between neighboring krill.

Schools of Antarctic krill were filmed with a stereophotogrammetry system comprised of two digital video cameras (Sony HDR-HC3E with 1600 × 896 pixel CMOS sensors) recording directly to digital video recorders at 25 frames per second. The two cameras were mounted 0.35 m apart on a moveable gantry platform located 1.25 m above the water surface. In order to provide a common viewing region, the cameras were angled inward at 30 degrees from the vertical, thus providing expected precisions of approximately 0.5 mm and 1.5 mm in the horizontal (*x* and *y*) and vertical (*z*) directions, respectively^35^. The camera system was calibrated by imaging a custom-built, immersed calibration device with 54 known coordinates distributed at four different elevations. A volume of approximately 37 cm (*x*) × 25 cm (*y*) × 59 cm (*z*) common to both cameras thus was calibrated. The water surface was undisturbed and remained at the same elevation throughout experiments and thus allowed high quality images to be acquired from both cameras. The cameras were synchronized via an LED light (blinking every 4.80 s) suspended beneath the gantry platform and visible in images from both cameras. Whereas the stereo camera system provided a high magnification view of a portion of the tank to provide measurements of krill positions, an identical third camera simultaneously recorded a low magnification oblique view of the entire tank surface to qualitatively determine the large-scale behavior of the entire school. In order to reduce visual cues that might disturb the krill^12,31^, the filming area surrounding the tank was draped in white plastic sheeting and the gantry platform was painted white.

**Figure 1:**
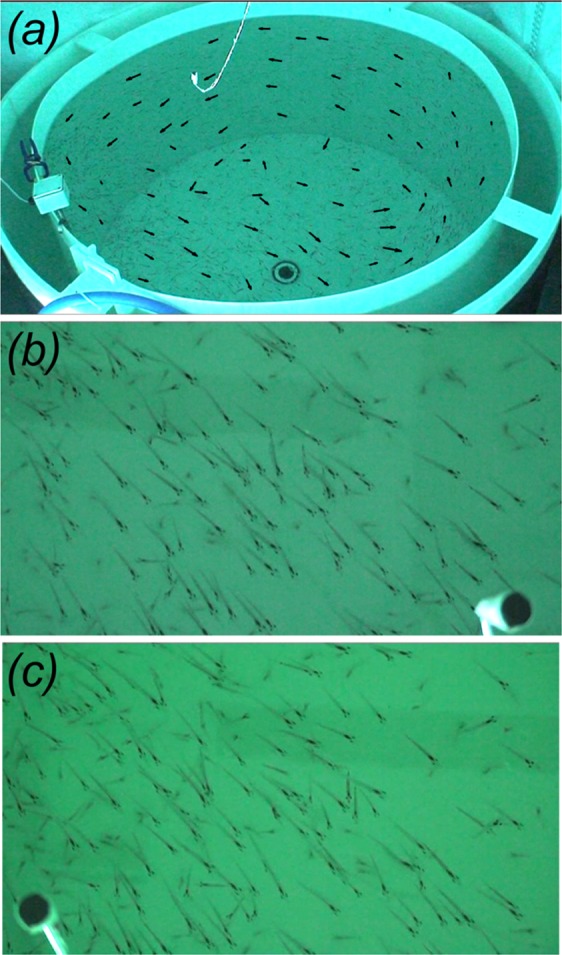
(**a**) Sample image from the overhead camera with an oblique view of krill schooling aquarium in which measurements were acquired. Vectors show the counterclockwise swimming directions of seventy-five krill selected and manually tracked to illustrate the large-scale motion of the school. (**b**) and (**c**) Sample synchronized images from the left and right video cameras in the stereophotogrammetry system, respectively.

Over the course of several days, the krill were filmed in the morning for several hours at a time and, as described by Kawaguchi *et al*., exhibited a variety of behaviors throughout the experiments, from random swarming to coordinated schooling^31^. The data set chosen for analysis was an 8 minute period in which the majority of krill strongly schooled around the tank periphery and in which the stereo cameras were properly positioned to capture this continuous stream of schooling animals. Figure 1a shows sample images from the overhead camera, and Fig. 1b,c show sample images from the left and right stereo cameras, respectively. Videos from the two stereo cameras were split into shorter segment pairs for further processing, and these segment pairs were temporally synchronized by the flashing LED using DLTdv5^36^.

Three-dimensional krill positions and headings were interrogated at 97 time points, each separated by at least 2 seconds to avoid repeatedly sampling the same group of animals. At each time point, three-dimensional krill positions were manually digitized using DLTdv5^36^. Krill positions in one to two frames before and one to two frames after the time point also were digitized to find the speed and heading vector of each animal. Thus, krill in five consecutive frames were tracked for 78 time points, and krill in three consecutive frames were tracked for the remaining 19 time points. Occlusions were not a major concern because of the transparency of the krill and because of the krill’s reluctance to swim directly beneath a conspecific. Because most krill swam horizontally during the experiments, the depth coordinate of each animal was averaged over the tracked frames to decrease positioning error in the *z* direction. The heading vector of each animal was defined as the vector from its position in the first frame to its position in the last frame. For time points at which five frames were tracked, the *z*-coordinates of the krill in the second and fourth frames were averaged with those in the first and fifth frames, respectively, to decrease error in the heading vector. An easily recognized point at the anterior tip of the gastric mill was chosen for digitization. Non-schooling krill near the tank bottom (i.e. far below the schooling animals near the surface) were not digitized. A few krill swimming below the school bulk, but above the tank bottom, were removed from the data set. The number of digitized krill at each time point ranged from 11 to 92 with a mean ± standard deviation of 63 ± 15. Following these procedures, a total of 5918 krill positions were determined.

Two different parameters describing group organization, polarity *P* and the polarization order parameter *O*_*P*_, were calculated so that krill school organization could be compared with fish school organization in both earlier studies (in which *P* is used) and more recent studies (in which *O*_*p*_ is more common). School polarity *P* was calculated both at each time point and globally (over all time points) as the mean of the deviation of each krill from the mean school direction at that time point^37^. Polarity falls within the range of *P* = 0–90° where a value of *P* = 0° corresponds to a perfectly aligned school and *P* = 90° corresponds to a disorganized school. The polarization order parameter *O*_*P*_ was calculated as the absolute value of the mean normalized individual krill heading and ranges between *O*_*P*_ = 0 for a random aggregation to *O*_*P*_ = 1 for a perfectly aligned school^38^. Individual swimming speed *V* was calculated by dividing the change in krill position between the first and last video frames at each time point by the elapsed time. Normalized swimming speed was calculated as *V*_*n*_ = *V/L*_1_. School density was calculated in each segment by dividing the number of krill present by the school volume. The alpha-shape algorithm was used to identify the points on the school boundary and to calculate the school volume interior to those points^30^. The alpha-shape algorithm used a sphere of radius 400 mm, a value chosen by visualizing the point cloud and its boundaries in three dimensions for different sphere radii and then selecting a radius that produced an accurate representation of the school.

**Figure 2:**
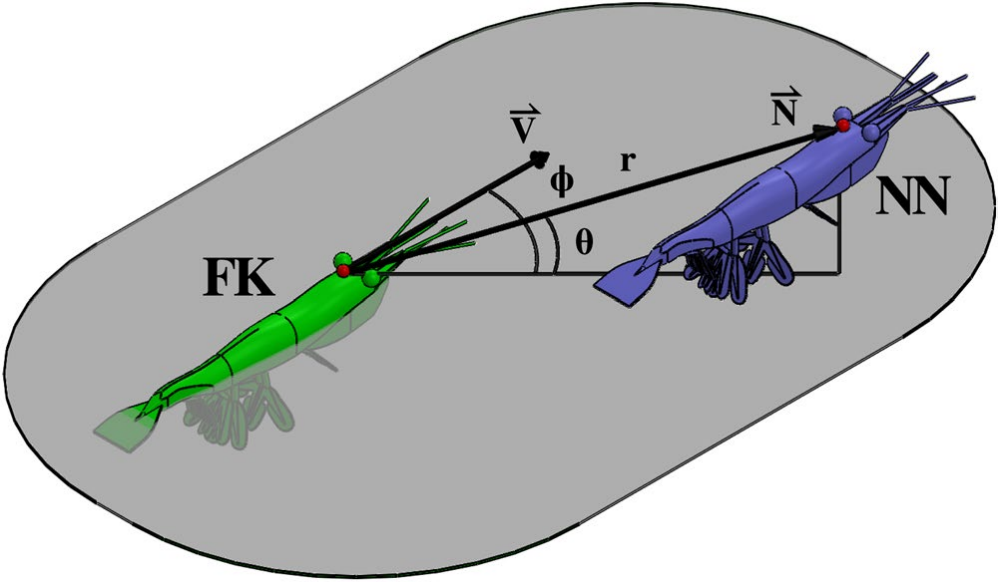
Three-dimensional illustration of a focal krill (FK) in green and its nearest neighbor (NN) in blue demonstrating the local spherical coordinate system with its origin at the focal krill’s gastric mill (red sphere). Both krill are swimming horizontally, but the NN is located at a higher elevation. The vector $\vec{V}$ represents the focal krill’s swimming direction and magnitude, and the vector $\vec{N}$ quantifies the distance and orientation from the focal krill to its nearest neighbor. The angle θ is the elevation angle and ϕ is the bearing angle of $\vec{N}$ in relation to $\vec{V}$, and *r* is the magnitude of $\vec{N}$ (i.e. the nearest neighbor distance).

The relative positions of krill were analyzed following the convention of the STARFLAG handbook^29,30,39,40^. Using the digitized point on the gastric mill as the origin, a local spherical coordinate system is defined by each krill’s heading vector $\vec{V}$ and its frontal plane. This coordinate system is established on each “focal” animal (Fig. 2). The swimming direction of each focal krill then is defined by θ = 0°, ϕ = 0°, where θ is the elevation angle corresponding to latitude (above or below the frontal plane) and ϕ is the (left-right) bearing angle corresponding to longitude. With each focal krill at the origin, the elevation angle θ, bearing angle ϕ, and distance *r* of the vector $\vec{N}$ to that animal’s nearest neighbor were found using custom Matlab code. A unit sphere with a representative focal krill at its origin was divided into 100 bins (bin dimensions of 18° in θ by 36° in ϕ), and, considering all focal krill at all time points, the number of nearest neighbors falling into each directional bin was tabulated. Dividing by the total number of nearest neighbor measurements gives a discrete probably density function. Further dividing by a probability density function describing a uniform (i.e. isotropic) point distribution produces the normalized angular density distribution of nearest neighbors, in which values greater than one indicate directions in which nearest neighbors are more likely to be found and values less than one indicate the opposite. In addition to analyzing the nearest neighbor position distribution (NN1), the distributions of second (NN2) and third (NN3) nearest neighbors also were analyzed.

Animals on aggregation boundaries only have neighbors in one direction and thus are often treated differently than interior animals in position analysis. For example, boundary animals may be allowed to serve as nearest neighbors of interior animals but not as focal animals themselves^30^. Such procedures help to prevent artefacts caused by the shape of the school from skewing nearest neighbor distance and position distributions. In the current system, interior and boundary krill were not differentiated in this way for two reasons. First, the krill school formed an annulus around the tank edge, but only a part of the school was imaged. Thus, the upper and lower school boundaries can be identified, but the constantly changing horizontal school boundaries usually extend beyond the imaged volume. Identifying true school boundaries is thus extremely difficult, if not impossible. The effect of conservatively treating all krill on the boundaries of the imaged volume as boundary krill (as determined by a convex hull or alpha-shape algorithm) and allowing them to serve as nearest neighbors but not as focal krill was tested. This procedure reduced the number of nearest neighbors available for position analysis to 3634; this reduction increased the noise in the distribution but did not significantly alter the key features of the nearest neighbor position distribution. Second, natural krill aggregations vary tremendously in size and shape and include the narrow, ribbon-type of schooling observed here in which a large percentage of animals would be classified as boundary krill^21,23^. Thus, we are justified in performing position analysis on all animals irrespective of their position within the school.

## Results

**Figure 3:**
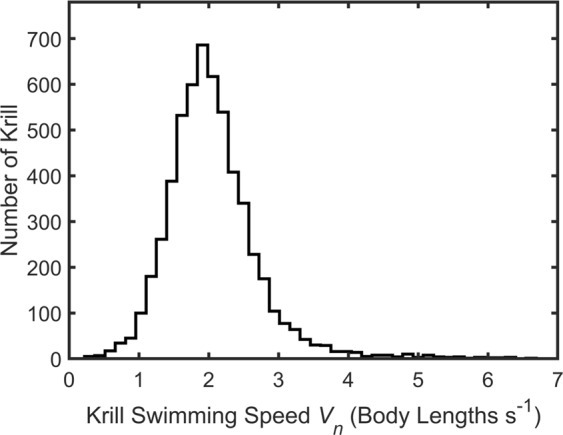
Histogram of normalized krill swimming speeds (*V*_*n*_ = *V/L*_1_), where *L*_1_ = 34 mm.

**Figure 4:**
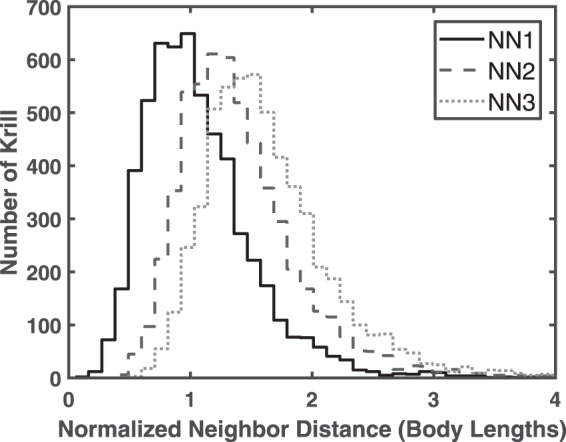
Histogram of nearest neighbor distances for first (NN1), second (NN2), and third (NN3) nearest neighbors normalized by *L*_2_, where *L*_2_ = 46 mm.

School polarity *P* across all time points was 34.0° and varied from 19.1° to 53.7° across the 97 individual time points. The mean polarization *O*_*P*_ was 0.78 ± 0.07 (mean ± standard deviation) and varied from 0.51 to 0.92 across video segments. Mean school density was 4244 ± 1726 animals/m^3^ (mean ± standard deviation) and varied from 2492–18860 animals/m^3^ across individual time points. Mean swimming speed over the first minute was 57.1 ± 16.5 mm/s (mean ± standard deviation), increased to 68.9 ± 28.7 mm/s (mean ± standard deviation) by the end of the second minute, and subsequently remained essentially steady, reaching 70.9 ± 21.3 mm/s (mean ± standard deviation) by the end of the video. Figure 3 shows a histogram of normalized animal swimming speeds. Overall mean swimming speed was 68 ± 24 mm/s (mean ± standard deviation) or 2.0 ± 0.7 body lengths per second (mean ± standard deviation) based on the mean body length of *L*_1_ = 34 mm. Median swimming speed was 65.0 mm/s. Based on the classification of Antarctic krill swimming modes by Murphy *et al*., most of the schooling krill are thus in the fast forward swimming mode, defined by a normalized swimming speed *V*_*n*_ = *V/L*_1_ greater than two body lengths per second^34^. Figure 4 shows histograms of the distance from the focal krill to its nearest (NN1), second nearest (NN2), and third nearest (NN3) neighbors normalized by body length *L*_2_ = 46 mm. Mean values of NN1, NN2, and NN3 are 47.1 mm, 63.0 mm, and 73.9 mm, respectively (a ratio of 1:1.34:1.57).

**Figure 5:**
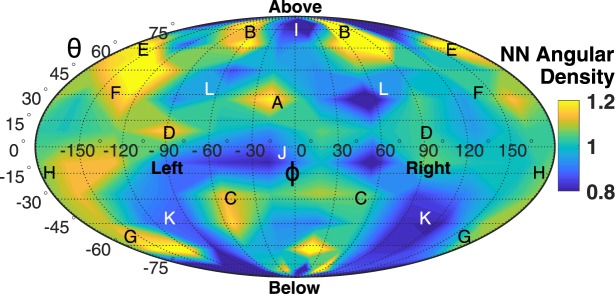
Contour plot of the angular density of nearest neighbors plotted on a Molleweide projection on which θ is the latitude and ϕ is the longitude. The swimming direction of the focal krill is θ = 0°, ϕ = 0°, and several directions (e.g. ‘Above,’ ‘Below,’ ‘Left,’ and ‘Right’) are indicated to help orient the reader. Warm colors indicate directions in which a nearest neighbor is more likely to be found, and cool colors indicate the opposite. Black letters A to H correspond to the likely nearest neighbor positions illustrated in Figs 6 and 7. White letters I to L correspond to unlikely nearest neighbor positions illustrated in Fig. 8.

**Figure 6:**
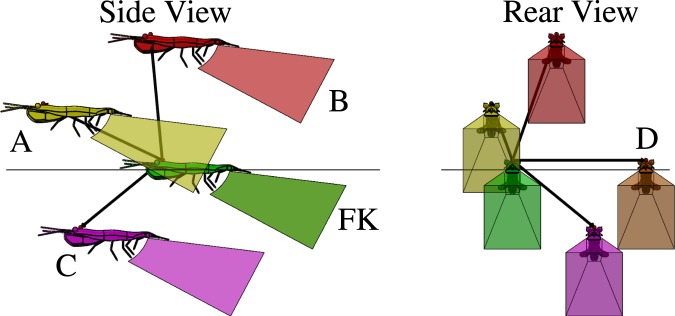
Lateral and posterior perspectives of the three-dimensional positions of four likely nearest neighbor positions (labeled A, B, C, and D corresponding to the hot-spot regions labeled in Fig. 5) relative to the focal krill (FK). Vectors ($\vec{N}$) of a magnitude equal to the mean nearest neighbor distance point from the focal krill to the respective krill A, B, C and D. The shapes behind each krill correspond to the volume occupied by the propulsion jet of an Antarctic krill engaged in fast forward swimming, as measured by Catton *et al*.^17^.

**Figure 7:**
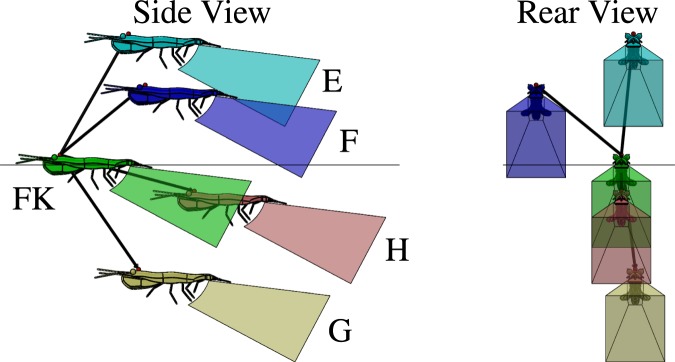
Lateral and posterior perspectives of the three-dimensional positions of four likely nearest neighbor positions (labeled E, F, G, and H, corresponding to the hot-spot regions labeled in Fig. 5) relative to the focal krill (FK). Vectors ($\vec{N}$) of a magnitude equal to the mean nearest neighbor distance point from the focal krill to the respective krill E, F, G, and H. The shapes behind each krill correspond to the volume occupied by the propulsion jet of an Antarctic krill engaged in fast forward swimming, as measured by Catton *et al*.^17^.

**Figure 8:**
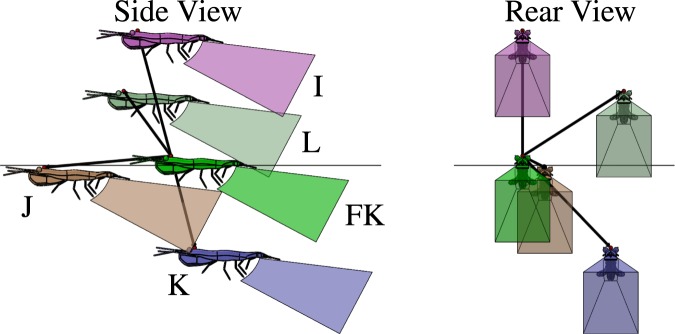
Lateral and posterior perspectives of the three-dimensional positions of four unlikely nearest neighbor positions (labeled I, J, K, and L corresponding to the cool-spot regions labeled in Fig. 5) relative to the focal krill (FK). Vectors ($\vec{N}$) of a magnitude equal to the mean nearest neighbor distance point from the focal krill to the respective krill I, J, K and L. The shapes behind each krill correspond to the volume occupied by the propulsion jet of an Antarctic krill engaged in fast forward swimming, as measured by Catton *et al*.^17^.

A contour map of nearest neighbor angular density projected onto a unit sphere using an area-preserving Mollweide projection^39^ is presented in Fig. 5. In this plot, the heading vector (i.e. swimming direction) of the focal krill is at θ = 0°, ϕ = 0°. Thus, a nearest neighbor at θ = 90°, ϕ = 0° would be located directly above the focal krill. Alternatively, a nearest neighbor trailing directly behind the focal krill would be located at both θ = 0°, ϕ = 180° and θ = 0°, ϕ = -180°. Warm colors (value >1) indicate regions in which a nearest neighbor is more likely to be found, whereas cool colors (value <1) indicate regions in which a nearest neighbor is less likely to be found. Black and white letters on the map correspond to likely (black) or unlikely (white) nearest neighbor positions. These positions are illustrated in Figs 6, 7 and 8 and will be discussed subsequently.

The angular density distribution in Fig. 5 is strikingly anisotropic and largely left-right symmetric about ϕ = 0°, thus mirroring the bilateral symmetry of the animal. Mirrored features in which a nearest neighbor is more likely to be found include the elongated regions marked ‘B,’ the inverted triangle region marked ‘C,’ and patches marked ‘E,’ ‘F,’ ‘G,’ and ‘H.’ Symmetric features in which nearest neighbors are less likely to be found include regions marked ‘L’ and ‘K.’ The unlikely regions form a roughly ‘figure eight’ shaped region centered at θ = 0°, ϕ = 0° in which the holes are the likely regions ‘A’ and ‘C’ and the apex is the unlikely region ‘I.’ Angular density values are greater on the left side of the focal krill than on the right. Rather than reflecting a preference of a focal krill to have its nearest neighbor on its left side, this asymmetry is an experimental artefact and is discussed subsequently.

Figures 6, 7 and 8 show the three-dimensional positions of likely (Figs 6 and 7) and unlikely (Fig. 8) nearest neighbors relative to a focal krill (FK) and will serve as the basis of the following analysis of the possible roles of hydrodynamic and visual cues in krill schooling. Each nearest neighbor krill is labeled corresponding to its position in Fig. 5. These illustrations use anatomically correct three-dimensional krill models with dimensions corresponding to the mean krill body length found in this study (*L*_1_ = 34 mm and *L*_2_ = 46 mm). Each krill is a distance of 47.1 mm from the focal krill, corresponding to the mean nearest neighbor distance measured in this study. The pyramid-shaped polyhedron behind each krill model represents the volume of the backwards-pointing jet-like flow generated by the metachronal beating of the krill’s pleopods during fast forward swimming^34^ as taken from the PIV measurements of Catton *et al*.^17^. As shown by Catton *et al*., this turbulent jet-like flow continues many body lengths downstream of the krill generating it^17^, but the polyhedron representing that flow in these diagrams is truncated for clarity. These diagrams thus provide realistic three-dimensional reconstructions of likely and unlikely krill positions within schools in relation to the flow produced by each animal and allow us to interrogate how hydrodynamic interactions among schoolmates may influence these positions. Ideally, the visual field of the focal krill also could be illustrated in Figs 6–8, but, to our knowledge, it has not been measured. However, it is known that euphausiids have large fields of view (e.g. 235° in *Meganyctiphanes norvegica*^41^) and may rotate their eyes 180° in order to follow a light source^24,42^. Further, since light direction is a strong orientation cue for Antarctic krill^24^, it is likely that the krill would look upward in response to the overhead light in the aquarium and thus have a field of view corresponding to at least their upper hemisphere (0° < θ < 90°, −180° < ϕ < 180°).

Figure 6 shows likely nearest neighbor krill positions (A, B, C, and D) in which the nearest neighbor is in front of or beside the focal krill relative to its swimming direction (a region defined by −90° < θ < 90°, −90° < ϕ < 90°). Position A is unique in that it is the only position in which the focal krill is in the propulsive wake of its nearest neighbor. Thus, the focal krill would receive hydrodynamic signals from a nearest neighbor at position A and is likely attracted to take up this position in its nearest neighbor’s wake. Minimal hydrodynamic interaction is expected between the focal krill and krill at positions B, C, or D. Positions A, B, and D are certainly within the field of view of the focal krill, but it is not clear whether the focal krill’s field of view extends downward to include position C. Further, positions A and C are the only positions in which the focal krill can be thought of as “following” its neighbor; that is, animals at positions A and C are “in front of” the focal krill and thus likely able to significantly influence its swimming direction. In contrast, neighbors in positions B and D (at approximately ϕ = ± 90°) advance concurrently with the focal krill (i.e. they are not “in front of” or “behind” the focal krill) and likely have a weaker influence on the focal krill’s swimming speed and direction.

Figure 7 shows likely nearest neighbor krill positions (E, F, G, and H) in which the nearest neighbor is behind the focal krill relative to its swimming direction (a region defined by −90° < θ < 90°, ϕ < −90°, ϕ > 90°). These nearest neighbor positions have less well-defined boundaries than positions A, B, or C, possibly indicating a weaker level of interaction between the focal krill and nearest neighbors “behind” the focal krill. The focal krill thus seems to interact most strongly with nearest neighbors ahead of it or to its sides. Positions E and F are likely within the visual field of the focal krill, but positions G and H are likely not. Position H is unique in that it is the only likely nearest neighbor position in the propulsive wake of the focal krill. The relationship between the focal krill and a krill at position H seems analogous to that between a krill at position A and the focal krill, with the focal krill switching from follower to leader. This relationship again indicates, this time from the perspective of the nearest neighbor, that Antarctic krill often occupy regions in which they receive hydrodynamic cues from anterior neighbors.

Figure 8 shows unlikely nearest neighbor positions I, J, K, and L relative to a focal krill FK. No hydrodynamic interaction is expected between the focal krill and its nearest neighbors at any of these positions. Position I represents an extremely low probability of having a nearest neighbor in that region and likely indicates a strong aversion of the focal krill to having its nearest neighbor directly overhead. A focal krill may avoid having its nearest neighbor in this position because the neighbor would block the overhead light that the focal krill needs to orient. If true, this may also explain the high likelihood of a neighbor being in region B (Fig. 6), because the focal krill may slightly change course to place the nearest neighbor in that region. Position J is almost directly in front of the focal krill, and the low probability of finding a nearest neighbor there may reflect the elongated shape of the Antarctic krill. The tail of a nearest neighbor in the region of θ = 0°, ϕ = 0° would almost touch the antennules of the focal krill, and the focal krill may prefer to maintain a greater distance to the animal directly anterior. The patches for positions L and K are large and diffuse in Fig. 5 and are located laterally at planes above and below the focal krill, respectively. Position L is likely within the focal krill’s field of view, whereas position K may or may not be. It is difficult to attribute the low frequency of these positions to a specific factor.

## Discussion

### Antarctic Krill School Characteristics

The characteristics of the Antarctic krill school measured in this laboratory study (e.g. speed, density, NND, and polarity) agree reasonably well with prior laboratory and field observations and are supported by a much greater number of observations. The mean school swimming speed measured here (6.8 cm/s or 2.0 body lengths/s) is approximately the same as that measured by Catton *et al*. for small groups of Antarctic krill swimming together (6.9 cm/s or 1.3 body lengths/s)^17^. The normalized swimming speeds also are similar once different measures of krill body length are considered. However, the school swimming speed measured here is less than the laboratory observations of Antarctic krill schooling by Kawaguchi *et al*. in which a mean swimming speed of approximately 20 cm/s was found^31^. Similarly, field observations by Kanda *et al*. showed schools of Antarctic krill swimming at an average speed of 14 cm/s^22^. Further, the krill swimming speeds measured here did not change greatly over the course of the experiment and are fairly tightly constrained around the mean, reflecting field observations of school segregation by size and swimming ability^21^.

Measurements of Antarctic krill school densities have varied greatly depending on the sampling technique, with some estimates reaching tens of thousands of krill per cubic meter^19,31,43^. The school density measured here (4244 krill/m^3^) does not reach this order of magnitude, but it is greater than the school density of approximately 1100 animals/m^3^ estimated by Kawaguchi *et al*.^31^. Our use of an alpha-shape algorithm to accurately define the school boundary likely contributed to this greater density measurement, but behavioral differences probably contributed as well since the school swimming speed in the current experiments was less than half that found by Kawaguchi *et al*.^31^. The mean NND is, of course, another way to look at school density. O’Brien^28^ found a mean NND of 0.6 body lengths, which is somewhat smaller than the currently measured mean NND of 1.0 body length, the NND of 3 body lengths found by Kawaguchi *et al*.^31^, and the NND of less than 2 body lengths found by Catton *et al*.^17^.

The organization level of Antarctic krill schools swimming in a flume was previously investigated by O’Brien^28^ as a function of environmental conditions such as water current and predation threat. For example, the krill were not organized without a current, but greater than 90% were parallel to their nearest neighbor at a weak current of 1.5 cm/s^28^. Unfortunately, the polarity metric used by O’Brien^28^ does not allow for direct comparison with the polarity or polarization order parameters used here. The currently measured polarity of 34° indicates a highly organized animal aggregation, and since measurements of group organization for aquatic invertebrates are lacking, we compare our measured values to those of fish schools. For example, Viscido *et al*.^44^ measured an average polarity of 39° for groups of four fish and 53° for groups of eight fish (*Danio aequipinnatus*), and Tien *et al*.^45^ measured polarity values of 63° without predators and 57° with simulated predator in a fish shoal comprised of two species. In addition, the currently measured mean polarization of 0.78 indicates group organization comparable to the mosquitofish schools studied by Herbert-Read *et al*., in which polarization values of 0.84, 0.71, and 0.63 were measured for schools of two, four, and eight fish, respectively^46^. Similarly, Chicoli *et al*. found median polarization values of 0.42 and 0.92 for schools of eight giant danio fish in still and flowing water, respectively^47^. Finally, Partridge *et al*. stated that the ratio of first, second, and third nearest neighbor distances served as a simple indicator of school structure, with values closer to one indicating greater structure^48^. The currently measured ratio of 1:1.34:1.57, which matches that found by O’Brien reasonably well (1:1.4:1.6)^28^, is also similar to that measured by Partridge *et al*. for saithe (1:1.3:1.5), a strong facultative schooler^48^. Antarctic krill and fish thus reach similarly high levels of organization when schooling.

The left-right asymmetry in the magnitude of angular density in the nearest neighbor position distribution (Fig. 5) is an experimental artefact reflecting the position, non-uniform density, and swimming direction of the krill school relative to the imaged volume. Specifically, the school mostly swims diagonally downward and to the right through the lower left side of the imaged volume, leaving the upper right side of the image much less populated (as in Fig. 1b,c). Thus, in the reference frame of the krill, the school’s right boundary (on the left and bottom sides of the image) is usually longer and more densely populated than the school’s left boundary (on the upper right side of the same image). A greater density of krill on the right boundary of the school translates into a greater proportion of nearest neighbors on the focal krill’s left^30^. The shape of the imaged volume also influences the nearest neighbor distribution because a krill in the lower left corner of the image can only have its nearest neighbor located on its left side. Nonetheless, the left-right asymmetry in Fig. 5 is largely a difference in magnitude and not in sign or position, thus giving confidence that the patterns observed are real. Further, this artefact represents a tradeoff between having a measurement volume large enough to observe the entire school (which would eliminate the artefact) and a volume small enough to determine the positions of individual krill with high certainty.

### Three-dimensional School Organization

Several mechanisms linking krill placement within schools to energy savings or hydrodynamic signaling have been proposed, but these hypotheses have sometimes suffered from a lack of knowledge of krill swimming hydrodynamics. For example, Wiese and Ebina hypothesized the krill propulsion jet as a communication channel linking schoolmates in a staggered diamond school structure^15,16,26^ but considered an unrealistic jet geometry in doing so. Later flow measurements support the idea that this jet could serve as a long-distance hydrodynamic signal, but a link between the jet and school structure has not been previously established^17,49^. In addition, Patria and Wiese^26^ hypothesized, based on flow visualization of a tethered krill, that krill could save energy by swimming in vortex rings surrounding their neighbor’s propulsion jet. However, because tethering an animal alters its appendage-generated flow field and creates flow patterns that differ from free-swimming specimens^50^, the vortices they observed are most likely artefacts of tethering. Subsequent flow measurements around freely swimming Antarctic krill, both hovering and swimming at speeds up to approximately 2 body lengths/s, have not revealed these vortex rings^17,51,52^. Instead, the rhythmic beating of Antarctic krill pleopods produces a pulsed jet that, with increasing swimming speed, breaks down into turbulent vortices. Other hydrodynamic mechanisms not based on vortices also may promote energy savings. For example, a novel energy-saving mechanism proposed here is based on the observation that a swimming krill carries a packet of fluid in its wake via Darwin’s drift^17,53,54^. A focal krill with a nearest neighbor at approximately θ = 0°, ϕ = 0° could potentially draft behind this neighbor while avoiding the backwards flow of its propulsion jet (which would increase the cost of swimming). Finally, these hypotheses should be considered against the null hypothesis that the nearest neighbor distribution is random or isotropic (i.e. that a neighbor is equally likely to be found in any direction).

The currently measured statistical distribution of nearest neighbor positions in combination with prior flow measurements of fast forward swimming Antarctic krill^17^ allow us to test these competing hypotheses. We consider first the energy-saving and signaling hypotheses and subsequently return to the null hypothesis when considering possible non-hydrodynamic effects on school structure. First, the hypothesis of energy savings via positioning in certain, consistently produced vortices^26^ is discounted for lack of experimental evidence that such flow structures exist for free swimming krill. However, future measurements at higher swimming speeds may reveal such a hydrodynamic mechanism, and the possibility of energy saving as a function of krill schooling is discussed later. Second, the nearest neighbor distribution shows no support for the theory that a focal krill attempts to save energy by drafting directly behind its nearest neighbor. Specifically, the nearest neighbor position distribution shows an angular density of less than unity in the region of θ = 0°, ϕ = 0°, indicating that this position is avoided. Third, the nearest neighbor distribution shows no support for the theory that a focal krill tends to avoid the backflow of its anterior neighbor’s posterior jet for energy savings. Instead, the nearest neighbor position distribution shows support for the hypothesis that a focal krill preferentially positions itself within the propulsion jet of its anterior neighbor for signaling purposes^16,17^. Specifically, a focal krill is within the propulsion jet of its anterior neighbor for θ and ϕ values of approximately -10° < θ < 10°, 10° < ϕ < 30°. As seen in Fig. 5, this zone has a nearest neighbor angular density of greater than unity and corresponds to position A illustrated in Fig. 6 and, considering the focal krill as the source of the jet, position H illustrated in Fig. 7. We thus conclude that Antarctic krill use the propulsion jet of their anterior neighbor as a hydrodynamic signal.

Other features of the nearest neighbor distribution found here likely result from factors unrelated to hydrodynamics. For example, the dearth of neighbors above a focal krill (region I) may result from a focal krill avoiding blockage of the overhead light needed for orientation. The high occurrence of neighbors in region B then may result from the focal krill slightly shifting position to keep region I clear of neighbors. In addition, vision plays a role in structuring the school, as shown by O’Brien’s finding that nearest neighbor distance increases with increasing light levels^28^. Tarling also found that high density swarms of krill are most common at night when the visual distance between neighbors is at a minimum^55^. We thus suspect that a focal krill, lacking a hydrodynamic connection with its nearest neighbors in regions B, C, and D, relies on vision for communication with these conspecifics and positions itself accordingly to sense changes in their speed or direction. However, further knowledge of the visual field and acuity of Antarctic krill is required to elucidate this connection and to determine the relative importance of visual and hydrodynamic cues under different environmental circumstances.

### Packing Effects

The high density packing of elongated animals into a school with a mean nearest neighbor distance of approximately one body length also likely affects the nearest neighbor position distribution. For example, the origin-to-origin distance from a focal krill to a nearest neighbor at its side (e.g. θ = 0°, ϕ = 90°) is likely smaller than for a nearest neighbor in front (e.g. θ = 0°, ϕ = 0°). Krill at ϕ = 90° would therefore be preferentially selected as nearest neighbors. The shape of closely packed animals may therefore impart structure to the nearest neighbor position distribution entirely apart from animal behavior^39,56^, and this apparent structure will vary as a function of animal shape and packing density. However, the nearest neighbor position distribution resulting from a random aggregation of these shapes cannot be found analytically as it can for a random aggregation of points (e.g. the uniform or isotropic case). Thus, the null hypothesis cannot truly be tested because the null nearest neighbor position distribution is not known. However, despite the potentially complicating effects of animal shape and packing density, three interlocking factors indicate with some certainty that the nearest neighbor position distribution measured here is not random. First, the left-right symmetry of many features in Fig. 5, which mirrors the animals’ bilateral symmetry, indicates school structure. Second, the high complexity of the nearest neighbor position distribution, with numerous regions in which nearest neighbors are more or less likely to be found, indicates a role for behavior. Third, if the effects of high density packing dominate the nearest neighbor position distribution, then top-bottom symmetry also would be expected. The absence of such symmetry may indicate the effect of animal behavior in response to conspecifics.

Animal shape and packing density also affect the angular density distribution in Fig. 5. The contour plot of nearest neighbor angular density in Fig. 5 was found by dividing the measured discrete probability density function by a theoretical probability density function describing an isotropic point distribution. In cases such as the current one in which the ‘points’ are actually closely spaced, interacting three-dimensional shapes, it would be more appropriate to divide by the (unknown) null distribution particular to the animal shape and packing density to determine which regions are more or less likely to have nearest neighbors. The angular density plot in Fig. 5 is thus likely a good first approximation of the true nearest neighbor position distribution but may overestimate or underestimate the likelihood of finding nearest neighbors in certain positions. The further development of tools to find null distributions for complex shapes packed at high densities is thus needed before it can definitively be stated that the nearest neighbor distribution found here is not random. Sensitivity studies also would be useful to determine the packing density at which animal shape may significantly impact nearest neighbor position distributions. For example, animal shape effects would likely dominate the nearest neighbor position distributions at the higher school densities observed by O’Brien^28^, in which mean nearest neighbor distances approach half a body length. Variations in krill body length also may affect interactions among conspecifics and the spatial structure of the school^21,57^.

### Energy Saving Hypotheses

Energy savings have long been suggested as a benefit of animal aggregations through a variety of aerodynamic or hydrodynamic mechanisms, and these benefits have usually required a particular geometric arrangement. For example, schooling fish were theorized to swim in a diamond configuration to save energy by two methods: the vortex hypothesis, in which fish position themselves within vortices that reduce the relative velocity of the oncoming flow; and the channeling hypothesis, in which the fish’s thrust is increased by the close proximity of its neighbors and their wakes^58,59^. Studies have not shown that fish take up the diamond lattice configuration required for the vortex hypothesis but have instead shown that fish derive an energetic benefit from schooling almost regardless of their position in the school^60–62^. Some of this benefit appears to be accrued from taking advantage of vortices^62^. However, Daghooghi and Borazjani showed that the large vortices created by the oscillating caudal fin required for the vortex hypothesis were quickly broken down in fish schools and thus not available for energy savings^59^. Instead, their study lent support to the channeling hypothesis, and a recent experimental study confirmed that fish save energy by swimming in a phalanx formation in which the channeling mechanism is evident^59,63^. Thus, in fish schools, while vortex energy recapture may play a role, the channeling mechanism seems to be dominant.

For krill schools, drafting, vortex energy recapture, and the channeling mechanism remain viable hypotheses for energy saving mechanisms. While the nearest neighbor position distribution measured here did not show evidence for drafting, a re-evaluation of the data taking into account the animal shape and packing density could change this assessment. In comparison with fish schools, the vortex hypothesis is considered unlikely to play a role in krill schools. Whereas a singly swimming fish oscillating its caudal fin leaves a well-organized wake comprised of large vortices that could conceivably be used for energy recapture, the wake of a swimming krill is created by five pairs of paddling pleopods that generate vortices that are small relative to the krill and thus presumably less useful for energy recapture. However, some synergistic interaction of these vortices may provide a useful benefit, and further experimental evidence is required. Lastly, the channeling mechanism, which has not been previously considered in relation to krill schooling, could potentially play a role in energy savings. A focal krill with a nearest neighbor in position H would find that neighbor acting as a lateral boundary on which its jet impinges, thereby potentially enhancing its own thrust. Further investigations ought to evaluate these three potential methods of energy savings in krill schools. An additional clue that krill schooling may serve an energy saving purpose is that tethered krill exposed to a pulsed hydrodynamic stimulus simulating a conspecific’s pleopod beat frequency were found to beat their pleopods at the same frequency^26^. Coordination of appendage strokes also indicates energy savings in fish schools^63^ and ibis formation flight^8^.

## Conclusions

Three-dimensional nearest neighbor statistics for Antarctic krill schools are provided in an effort to determine how school structure relates to energy saving or intra-school communication benefits. The krill schools reach similarly high levels of organization as fish schools while swimming at speeds of two body lengths per second at nearest neighbor distances of one body length. The results support the idea that behavioral or geometric factors, including energy savings, avoidance of light-blocking overhead neighbors, and the elongated body shape of Antarctic krill, may play a role in determining the three dimensional spatial structure of tightly packed krill schools. The measured nearest neighbor position distribution indicates that Antarctic krill within schools preferentially position themselves within the propulsion jet of the nearest neighbor swimming ahead of them. This position allows krill to gather information about the swimming of their anterior neighbor via hydrodynamic signals within the jet. The pulsed propulsion jet, created by the metachronal stroking of the neighboring krill’s pleopods, thus comprises an unsteady hydrodynamic communication channel between neighbors that structures the school.

## Data Availability

Data will be provided upon reasonable request.
